# Endophytic fungi specifically introduce novel metabolites into grape flesh cells *in vitro*

**DOI:** 10.1371/journal.pone.0196996

**Published:** 2018-05-07

**Authors:** Li-Hua Huang, Ming-Quan Yuan, Xiu-Jin Ao, An-Yun Ren, Han-Bo Zhang, Ming-Zhi Yang

**Affiliations:** 1 School of life science, Yunnan University, Kunming, China; 2 School of chemistry and chemical engineering, Yunnan University, Kunming, China; Tallinn University of Technology, ESTONIA

## Abstract

Since endophytes can affect metabolism of host plants, they are expected to be used to improve crop quality, especially for crops with organoleptic sensitive products such as wine grape. However, details of metabolic interactions between endophytes and host plants were less understood. In this work, we used high pressure liquid chromatography (HPLC) to analyze the metabolites of fruit flesh cells of grape treated with dual culture of different endophytic fungal strains (EFS). We observed that the dual-culture with different fungal strains show different metabolites composition in grape cells. In response to different EFS, quantities of detected metabolites in grape cells varied from 6 to 17 in this assay, and 1 to 11 novel metabolites were introduced into metabolome of grape cells. Dual-culture with fungal strains CS2, RH16 and RH5 introduced the highest quantities (10 or 11) of novel metabolites in grape cells. More importantly, the modification of metabolic profiles in grape cells *via* fungal endophytes appeared to be fungal strain/genus-specificity. Overall, this work revealed that introduction of specific metabolites in host plants may be one consequence during the process of endophytes-host metabolic interactions, which raise the possibility to shape grape qualities and characteristics using tool of fungal endophytes.

## Introduction

Endophytes were intensively studied during the past decades for the great potential of novel valuable metabolites which have medicinal, agricultural and industrial applications [1–5]. However, despite numerous reports, there still no major breakthroughs in terms of commercial exploitation of any endophytes as a source of bioactive molecules [6]. The symbiosis of endophytes conferred growth promotion and environmental adaptability effects to host plants on the other hand, have achieved applications [7–9]. The fact that the endophytes had metabolic impacts on host plants, further suggests the possibility of regulating the biochemical status of host plants with fungal endophytes [10]. This may be of great interest to food crops which give organoleptic sensitive products such as wine, coffee and tea. Therefore, clarifying the mechanisms undergo endophytes-hosts metabolic interaction is fundamental for this purpose. Studies concerned the interactions between endophytes and host plants had been documented [9, 11, 12], while the metabolic contributions of these endophytes to their host plants is less covered so far. Due to the complexity and difficulty to investigate the metabolic interactions between endophytes and their host plants *in vivo*, we instead analyzed metabolome of *in vitro* plant cells and dual culture system.

Dual culture has been successfully used in studying the physiological and morphological interactions between fungal endophytes and plant cells [13–15]. Previously, fungal endophytes were classified into categories based on their infective and detrimental abilities to the grape cells in dual cultures [13], but how these fungal strains furtherly influence the metabolites of grape fruit cells are expected. This report, however studied the impacts of multiple endophytic fungal strains on the metabolite profiles during the dual culture and provided some clues for the mechanism of plant cell-endophytes metabolic interactions.

## Materials and methods

### Grape calli preparation

Cell line (CBL, kindly provided by Professor Serge Delrot, director of Research Lab. of grapevine physio-ecological and functional genomics, France) induced from flesh of young grape berries (*Vitis vinifera*, cultivar: Cabernet sauvignon) was used in this study. B5 solution with 3% sucrose, 0.2 mg/L cytokinin, 0.1 mg/L naphthylacetic acid (NAA) and 0.8% agar was prepared as callus medium for callus sub-culture and the following dual culture with fungus. Prepared grape calli for this experiment were in the logarithmic growth phase.

### Preparation of endophytic fungal strains

Foliar endophytic fungal strains (EFS, Table 1) were isolated from grape variety ‘Cabernet sauvignon’ (*Vitis vinifera*) and another local variety, ‘Rose honey’ (*V*. *Vinifera L*.*× V*. *labrusca L*.) in local vineyards (Yunnan province, China) (Table 1). Vineyards located in subtropical climate area, within the latitude from N26 to N27, and altitude from 1400 to 2500 m. Endophytic fungi isolation followed the tissue patch method [16], with some modifications. Briefly, healthy leaves (grapevine) without any symptoms of disease were chosen for EFS isolation. Leaves were done surface sterilization with the procedure of 75% ethanol, 1 minute; 3% sodium hypochlorite, 20 minutes; and washed 3 times in sterilized water. The surface sterilized leaves were then cut into 0.5×0.5 cm patches and pasted on potato dextrose agar (PDA) medium (containing 50 mg/L of carbenicillin and streptomycin) in petri dishes. Plates were incubated at 25 °C and examined the emergence of fungi every day. The emerged fungi were transferred to PDA plates to obtain pure cultures. Prior to initial plating, several samples were imprinted onto media and these imprinted plates were monitored for the lack of fungal growth to ensure the effectiveness of the surface sterilization. Pure cultured endophytic fungal strains were identified using ITS DNA sequences (Ma et al., 2014). Before performing dual culture with grape cells, fungal strains were plate cultured on potato dextrose agar (PDA) medium in petri dishes for one week.

**Table 1:** Endophytic Fungal strains (EFS) used in the experiment.

Strain ID	Species	Strain ID	Species
RH5	*Trichothecium sp*.	RH38	*Epicoccum nigrum*
RH6	*Alternaria alternaria*	RH43	*Alternaria arborescens*
RH7	*Epicoccum nigrum*	RH44	*Alternaria arborescens*
RH12	*Niqrospora sphaerica*	RH45	*Epicoccum sp*.
RH16	*Alternaria sp*.	RH46	*Niqrospora sp*.
RH24	*Alternaria arborescens*	RH48	*Colletotrichum gloesporioides*
RH28	*Alternaria alternaria*	CS2	*Colletotrichum gloesporioides*
RH31	*Alternaria alternaria*	CS11	*Epicoccum nigrum*
RH32	*Alternaria alternaria*	CS13	*Fusarium oxysporum*
RH34	*Trichothecium roseum*	CS16	*Alternaria sp*.
RH37	*Epicoccum nigrum*		

Fungal strain ID with ‘RH’ represents the endophytic fungal strain was isolated originally from grape cultivar Rose honey (*Vitis*. *Vinifera* L.× *V*. *labrusca* L.), and with ‘CS’ means the fungal strain was isolated from another grape cultivar Cabernet sauvignon (*V*. *vinifera* L.).

### Establishment of fungi-calli dual-culture system

Sterilized 30 mL callus medium was added to each sterilized petri dishes to generate solid culture plates. Dual cultures were performed as described by Huang et al. (2017). Calli without fungal inoculation were used as callus control. Every treatment and control contains more than 3 biological replicates.

### Sample harvest and pre-treatment

Calli were harvested after 10 days dual-culture with fungi. All harvested grape calli were grounded into powder with liquid nitrogen and then freezing dried in a vacuum freeze dryer.

### HPLC assay for methanol extracts of grape cells

Ten milligram of freezing dried grape callus powder were accurately weighed and extracted with 500 μL of 60% methanol (contains 0.1% of hydrochloric acid) for 2 hours in an ultrasonic cleaner. Extracts were centrifuged briefly at 10000 rpm and supernatants were then filtered with 0.45 um filter columns. Ten micro liter of extracts were loaded and metabolites in grape cells were separated by reverse C18 column on a HPLC instrument (Agilent, USA) with 30 °C of column temperature. Elution phase is acetonitrile: water: formic acid = 35: 65: 0.1, with the elution speed of 1 mL/min, and detected with a UV detector at 254, 263 and 280 nm, respectively. Samples were eluted with the gradient procedures as illustrated in supplementary table (S1 Table).

### Data analysis

UV detector at 254 nm detected the most metabolites in this HPLC assay, and data acquired at this detection wavelength were used in this analysis. Metabolome between treatments were directly compared the chromatograms. Peak areas were used to compare the relative contents of certain detected compounds in HPLC, and reported as means ± standard deviation of all replicates. And the statistical differences among treatments were determined using one-way ANOVA followed by Tukey’s test (P<0.05) on SPSS16.0 (SPSS Inc., Chicago, IL, USA). The confirmation of certain metabolites in one treatment determined only when this compounds appeared in two third of the replicates. Treatments were done Squared Euclidean distance Hierarchical clustering using SPSS 16.0 software, based on the appearance (1) and absence (0) matrix of all detected metabolites in grape cells. Heat-map were generated (on excel 2013) according to the mean peak areas of the appeared metabolites.

## Results

**Fig 1:**
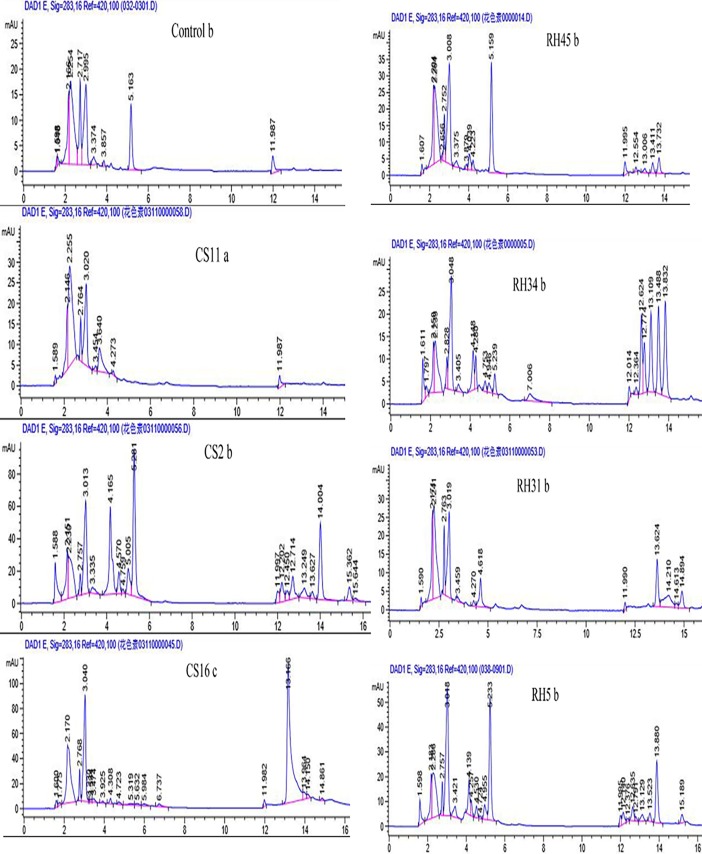
HPLC chromatograms of grape cell extracts after dual-cultured with different endophytic fungal strains (EFS). Chromatograms were selectively displayed in the figure. Each chromatogram was marked the EFS which the grape cells dual-cultured, and the followed letter was the serial number of replicates. Chromatograms of all other detected samples can be found in supplementary materials (S1 Fig).

HPLC clearly displayed the differences of metabolite profiles in grape flesh cells after the dual-culture with fungal endophytes (Fig 1). Dual-culture with different endophytic fungal strains (EFS) led to the establishment of different metabolites patterns (Fig 1). In this HPLC assay, the detected metabolites mainly appeared within retention time from 2.0 to 16.0 minutes and obviously separated into two clusters, one appeared at retention times between 2.0 and 5.0 minutes, whereas the other appeared at retention within 12.0 and 14.0 minutes (Fig 1). In comparison with the control, dual-culture with EFS RH5, RH16, RH34 and others has robustly increased numbers of detected metabolites in grape cells (Fig 1, S1 Fig).

**Fig 2:**
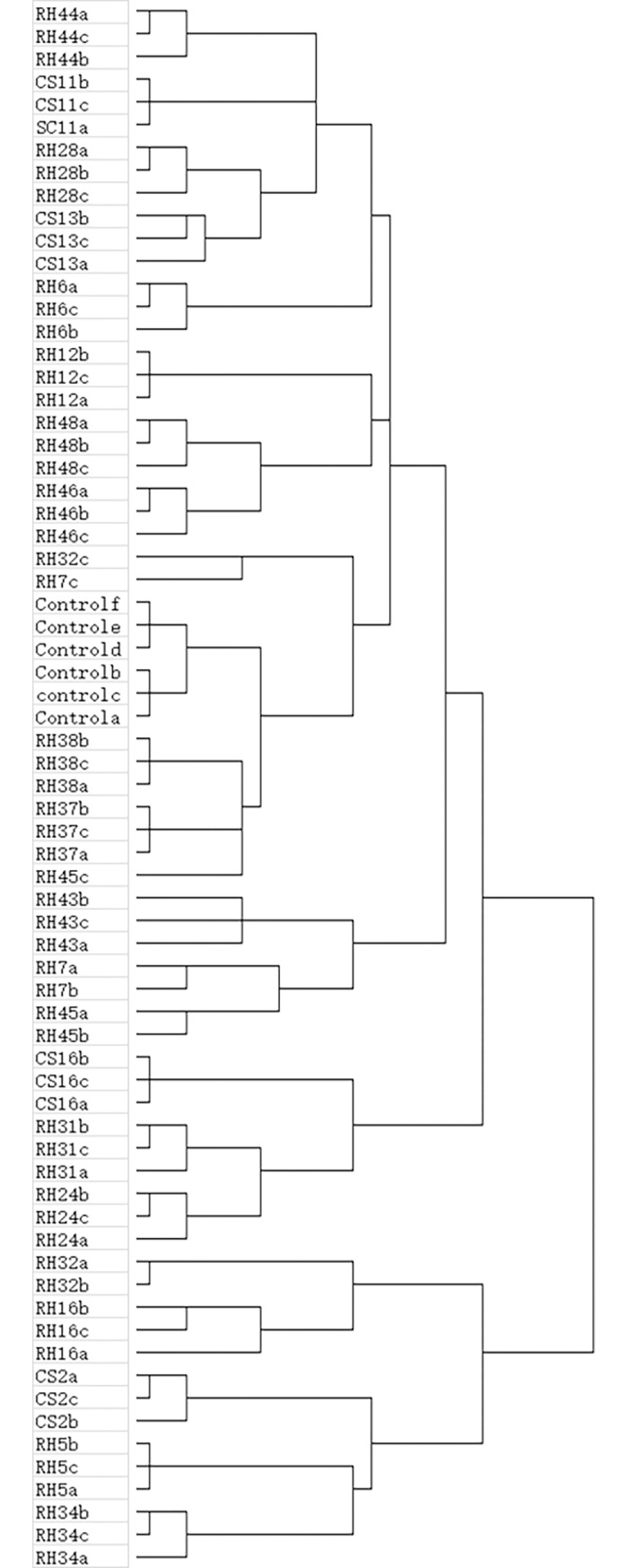
A clustering (Squared Euclidean distance Hierarchical clustering using SPSS 16.0 software) to all replicates of the treatments based on the appearance (1) and absence (0) matrix of detected metabolites.

Clustering to all samples based on the presence (1) and absence (0) matrix of metabolites, biological replicates of one treatment tend to be clustered together with few exceptions (Fig 2), suggesting that metabolite change in grape cells by fungal endophytes are reproducible and EFS-specific. Exceptions were found in grape cells after co-cultured with three EFS: RH45, RH7 and RH32. Dual-culture with these EFS make the metabolites composition in grape cells varied among replicates (Fig 2).

**Fig 3:**
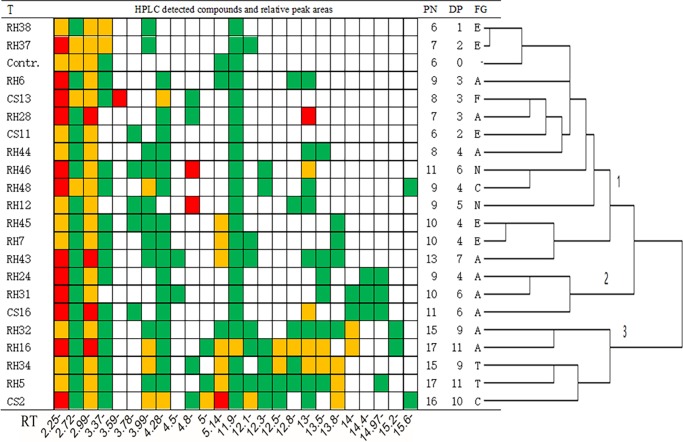
HPLC detected metabolomes and metabolites contents in grape cells, as well as the clustering to all treatments. T: treatment (represent as endophytic fungal strain ID and the control (Contr)). HPLC detected compounds are marked as colored bricks, and different color represent the relative content (peak area) of the metabolites: 10 mAU*S ≤green <100 mAU*S; 100 ≤yellow < 500 mAU*S; red ≥ 500mAU*S. PN: peak numbers; DP: novel peak numbers when compared to the control; FG: genus of the EFS, E: *Epicoccum*; A: *Alternaria*; F: *Fusarium*; N: *Niqrospora*; C: *Colletotrichum*; T: *Trichothecium*. At the bottom of the figure displayed the retention time (RT) at which the metabolites appeared in this HPLC assay.

Fig 3 summarized the HPLC detected metabolite compositions, peak numbers and relative contents of these detected compounds in grape cells under different treatments, as well as the clustering of all those treatments in the experiment (Fig 3). In total 25 metabolites were detected in this assay and numbers of the detected metabolites in grape cells of different treatments varied from 6 to 17 (Fig 3). EFS CS2, RH16 and RH5 dual-cultured grape cells detected the highest quantities (16 or 17) of metabolites, whereas in EFS RH38, RH37, RH28, CS11 dual-cultured grape cells and controls detected less quantities (6 or 7) of metabolites (Fig 3). In comparison with control, 1 to 11 novel metabolites were produced in grape cells due to the existences of different EFS in the dual culture. Accordingly, dual culture with EFS CS2, RH16 and RH5 introduced the most quantities (10 or 11) of novel metabolites, whereas, dual culture with EFS RH38, RH37 and CS11 induced the least quantities (1 or 2) of novel metabolites into grape cells (Fig 3, S2 Table).

Apart from the metabolite at retention time of 3.59 minutes which only appeared in fungal strain CS13 (*Fusarium sp*.*)* dual-cultured grape cells, all other metabolites in this HPLC assay can be detected in two or more EFS treated grape cells (Fig 3). Notably, three specific metabolites at retention times of 4.5, 4.8 and 15.6 minutes were detected, and each of these metabolites was only present in two of the used EFS dual-cultured grape cells (Fig 3). Fungal strains RH31 and RH43 which introduced the metabolite at retention time of 4.5 minutes belong to same genus *Alternaria*. Co-culture with EFS RH48 and CS2 which from the genus *Colletotrichum* produce the metabolite at retention time of 15.6 minutes in grape cells. And additionally, EFS RH46 and RH12 initiated higher relative content of metabolite (peak areas>500 mAU*s) at retention time of 4.8 minutes, and these fungal strains also belong to the same genus *Niqrospora* (Fig 3, S2 Table).

Clustering analysis based on the metabolites patterns in grape cells, all EFS and control can be categorized into 3 groups. EFS RH37, RH38, RH6, CS13, RH28, CS11, RH44 were closely clustered with control (group 1), suggested the less metabolomics impacts of these EFS on grape cells (Fig 3). Except the control, fungal treatments in group 1 included all of the EFS from genus *Epicoccum* (5/5: five of five used EFS in this experiment), *Niqrospora* (2/2), *Fusarium* (1/1), and half of the EFS from genus *Alternaria* (4/9). Group 2 clustered with 3 EFS, and all these fungal strains belong to genus *Alternaria*. The left group contain 5 fungal strains, included two EFS from genus *Trichothecium* (2/2) and *Alternaria* (2/9), respectively, and another fungal strain CS2 from genus *Colletotrichum* (Fig 3). No obvious differences were observed in this experiment of the metabolic effects on grape cells between host and non-host isolated EFS (Fig 3).

Dual-culture with fungal endophytes not only modified the composition of metabolites, but also influenced the relative contents of the co-detected metabolites in the grape cells. Five metabolites at retention times of 2.25 (metabolite A+B), 2.72 (C), 2.99 (D), and 11.9 (E) minutes in this assay, were detected in all samples (Figs 1 and 3 and S2 Table). Relative contents (represent as peak areas mAU*S) of these metabolites and different significances among treatments were presented in Table 2. Relative content of these metabolites in grape cells were obviously varied due to the dual-cultured EFS, and some of these changes have reached the statistical significance (Table 2). For some examples, compared to the control, dual-culture with EFS RH16 and CS16 significantly promoted the content of metabolite C, whereas dual culture with another EFS CS11 suppressed the content of this metabolite (Table 2). Dual culture with EFS RH16 also significantly promoted the content of metabolite E in grape cells (Table 2).

**Table 2:** Peak areas (mAU*S) of co-detected metabolites in grape cells and the different significances.

Compound	A+B (RT = 2.25)	C (RT = 2.73)	D (RT = 2.99)	E (RT = 11.9)
Control	314.45±52.85a*	131.33±54.54a	165.89±23.93cd	29.61±1.10b
11RH6	967.31±699.53a	75.14±33.32a	259.95±87.09abcd	17.39±7.79b
RH12	449.95±83.03a	78.65±26.06a	319.30±7.35abcd	11.02±1.79b
RH28	868.14±204.53a	78.24±8.00a	576.35±190.93abc	14.97±7.83b
RH32	375.67±133.15a	55.19±20.49a	308.81±25.05abcd	35.06±27.29b
RH34	321.56±148.48a	43.81±11.18a	400.04±210.50abcd	19.27±3.74b
RH37	592.72±643.11a	206.92±238.69a	273.37±242.95abcd	28.01±3.27b
RH38	460.06±176.15a	87.38±67.97a	178.45±67.69cd	21.05±1.89b
RH45	336.72±51.78a	81.13±22.33a	216.33±66.79abcd	26.84±4.06b
RH46	646.90±25.72a	85.54±32.38a	390.41±55.22abcd	15.27±5.28b
RH48	1176.38±402.78a	128.10±43.79a	363.04±36.75abcd	42.00±18.22b
CS2	553.15±75.85a	59.96±4.81a	487.96±48.52abcd	48.78±23.92b
CS11	342.38±66.18a	54.78±13.84a	137.29±24.08e	15.84±3.98b
CS13	1100.10±570.09a	220.35±134.67a	361.06±246.69abcd	30.71±10.51b
CS16	1203.52±456.04a	70.66±18.90a	621.24±36.69a	26.20±0.92b
RH5	384.48±54.94a	58.72±3.85a	404.94±53.19abcd	26.26±2.01b
RH7	400.32±13.41a	97.15±11.74a	307.72±71.02abcd	15.65±3.15b
RH16	593.52±261.40a	73.46±33.72a	611.46±293.05ab	105.13±70.65a
RH24	695.25±391.96a	79.42±23.05a	198.62±65.76bcd	20.28±5.79b
RH31	926.19±465.05a	81.08±12.26a	258.50±91.48abcd	10.78±1.03b
RH43	696.70±17.33a	79.55±4.51a	508.87±250.12abcd	15.67±2.59b
RH44	247.98±58.41a	52.14±8.83a	197.52±29.39 23bcd	26.60±19.12b

Values were displayed as means of all replicates ± standard variations.

*Letters indicate the different significances of values within columns. And values followed with totally different letters are significantly different (P≤0.05).

## Discussion

Metabolites in wine grape berries contribute most to wine qualities and characters [17]. How abiotic and biotic environmental factors such as temperature, water conditions, radiations, pathogens and others on wine grape chemistry and their resultant wines were intensively studied [18–22]. However, little is known how endophytes, one of biotic environmental factors, affect metabolism on grapevine and other plant. Endophytes had been proven to participate in the plant growth and stresses adaptability [7–9]. And all these changes in growth and adaptability for host plants were fundamentally the alteration of plant metabolisms. Re-inoculation of fungal endophytes to field grow grapevines had significant metabolic impacts on grape leaves and fruits, suggested the possible application of endophytes in grape quality regulation [10]. In fact, results in this field experiment integrated effects of exogenous fungal inoculation and the inoculation interfered endogenous endophytes communities. Our current report using dual culture system, which allowed to furtherly analyze the metabolic changes by certain EFS along on grape cells. Previous dual culture of these EFS with grape cells (shoot induced cells) had categorized fungal strains due to their specific interactions [13]. It will be of great interest if EFS also impose specific metabolic impacts on grape flesh cells.

Even not qualitatively identified all those detected metabolites in this HPLC assay, results of this assay were enough to confirm the significant effects of fungal endophytes on the metabolome of plant cells. Novel metabolites were produced as well as the relative contents of metabolites in grape cells were modified due to the existence of fungal endophytes (Fig 1, Table 2). Because of the symbiosis of fungal endophytes, similar circumstances may occur in natural growing plants *in vivo*. As expected, strain-specific fungal endophytes on grape cellular metabolome modification was observed (Fig 2, S1 Fig). Certain degrees of genus-specificity, however, was also detected in fungal endophyte-grape cell metabolic interactions (Fig 3). EFS belong to the same genus such as *Epicoccum*, *Niqrospora* and *Alternaria* tend to clustered together (Fig 3). And specific metabolites were only detected in grape cells dual-cultured with certain genus of fungal endophytes (Fig 3). Therefore, beside the specific morphological interactions[13], specific metabolic interactions also occur between fungal endophytes and grape cells. This will be important in guiding the selections of candidate EFS for purpose shaping the qualities and the characteristics of grape in viticulture.

Generally, mechanisms underlying the metabolic impacts of endophytes on host plant may employ pathways of: i) endophytes self-metabolizing; ii) endophytes and host co-metabolizing; and iii) Signaling [23]. The fact that novel metabolites were introduced into grape cell which has been confirmed in this work, implies the significant pathways of endophytes self-metabolizing and endophytes-host co-metabolizing during the endophyte-host metabolic interactions. But details on how certain novel metabolite produced and appeared in plant cells during the process of endophytes symbiosis need further researches.

In conclusion, the introduction of novel metabolites may be one mechanism that endophytes influence host plant’s metabolism. And these newly produced metabolites may employ pathways of endophytes self–metabolizing or endophytes and host co-metabolizing. The specific effects of endophytic fungal strains on grape cellular metabolites composition raise the possibility of purpose shaping of grape qualities and characters in viticulture by means of endophytes.

## Supporting information

S1 Fig HPLC chromatograms of all samples of grape cell extracts after co-cultured with different endophytic fungal strains (EFS). (PDF)

S1 Table Approaches gradient elution for HPLC. (PDF)

S2 Table HPLC detected metabolites and peak areas (mAU*S). (PDF)
