# Supplementary material for: Endophytic fungi specifically introduce novel metabolites into grape flesh cells in vitro
Source: PLoS One. 2018 May 7;13(5):e0196996. doi: 10.1371/journal.pone.0196996 (PMC5937782; doi:10.1371/journal.pone.0196996)
Supplement: S1 Fig — (PDF) [file pone.0196996.s001.pdf]

S1 fig

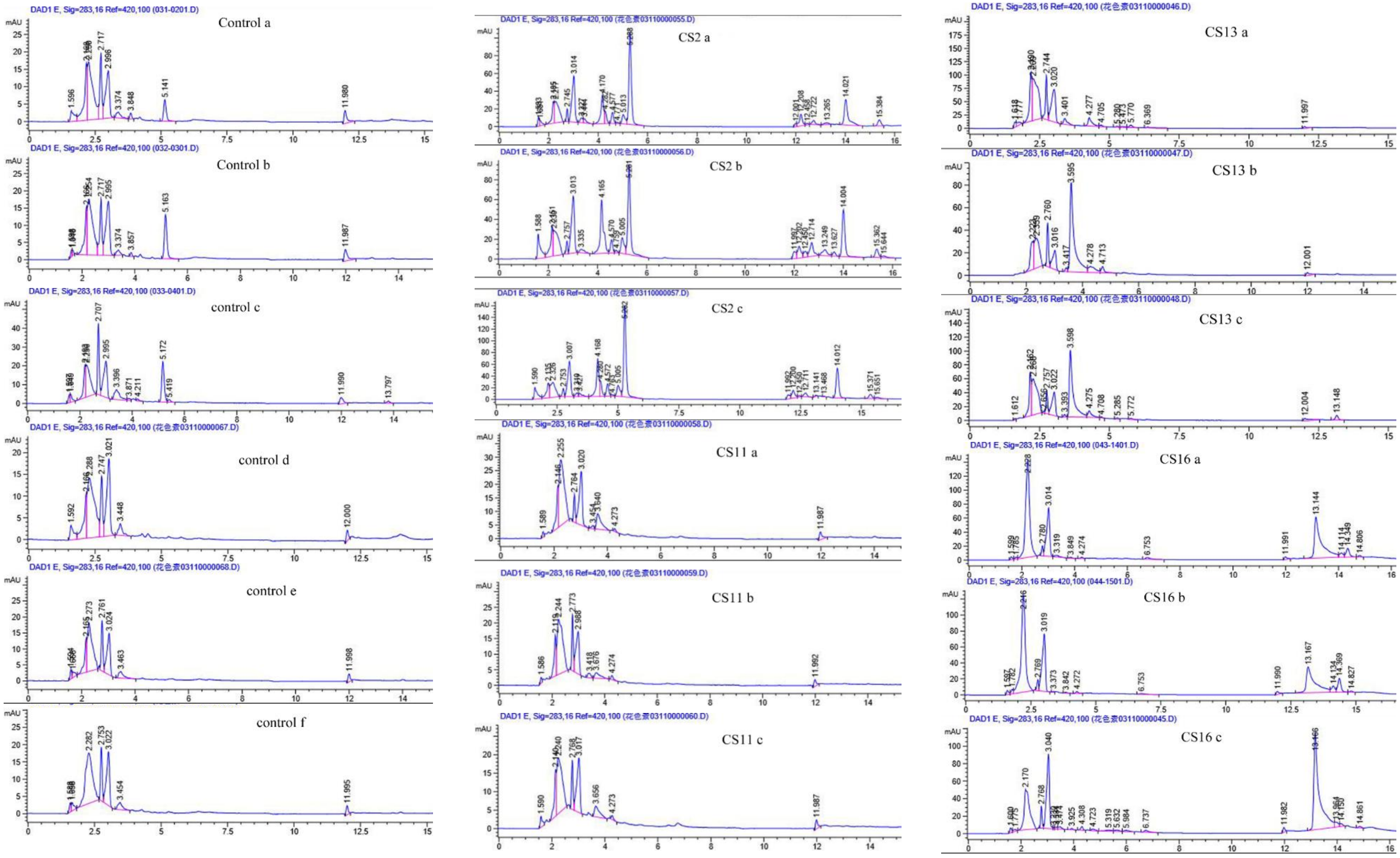
Control a
Control b
control c
control d
control e
control f
CS2 a
CS2 b
CS2 c
CS11 a
CS11 b
CS11 c
CS13 a
CS13 b
CS13 c
CS16 a
CS16 b
CS16 c

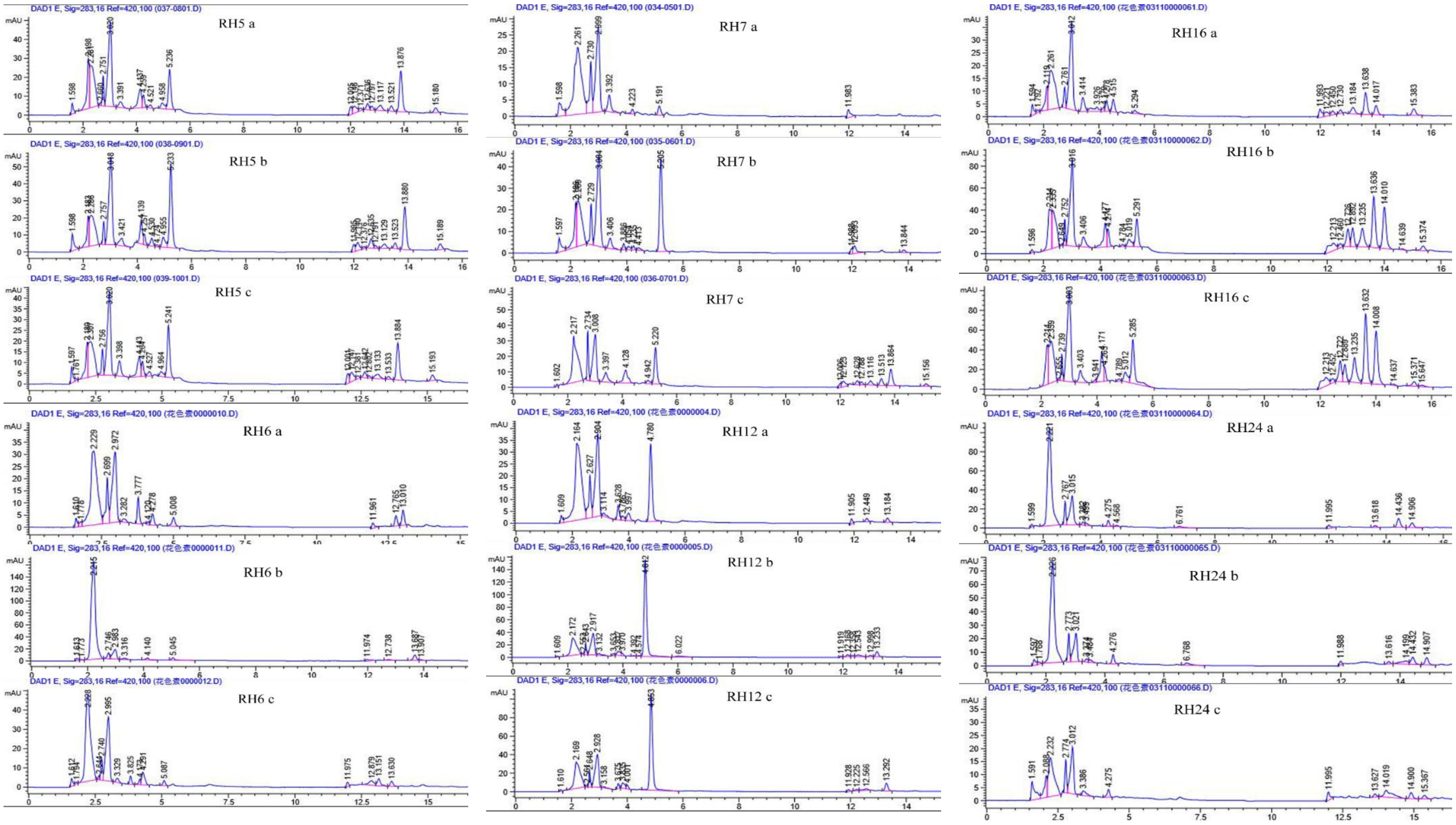
RH5 a
RH5 b
RH5 c
RH6 a
RH6 b
RH6 c
RH7 a
RH7 b
RH7 c
RH12 a
RH12 b
RH12 c
RH16 a
RH16 b
RH16 c
RH24 a
RH24 b
RH24 c

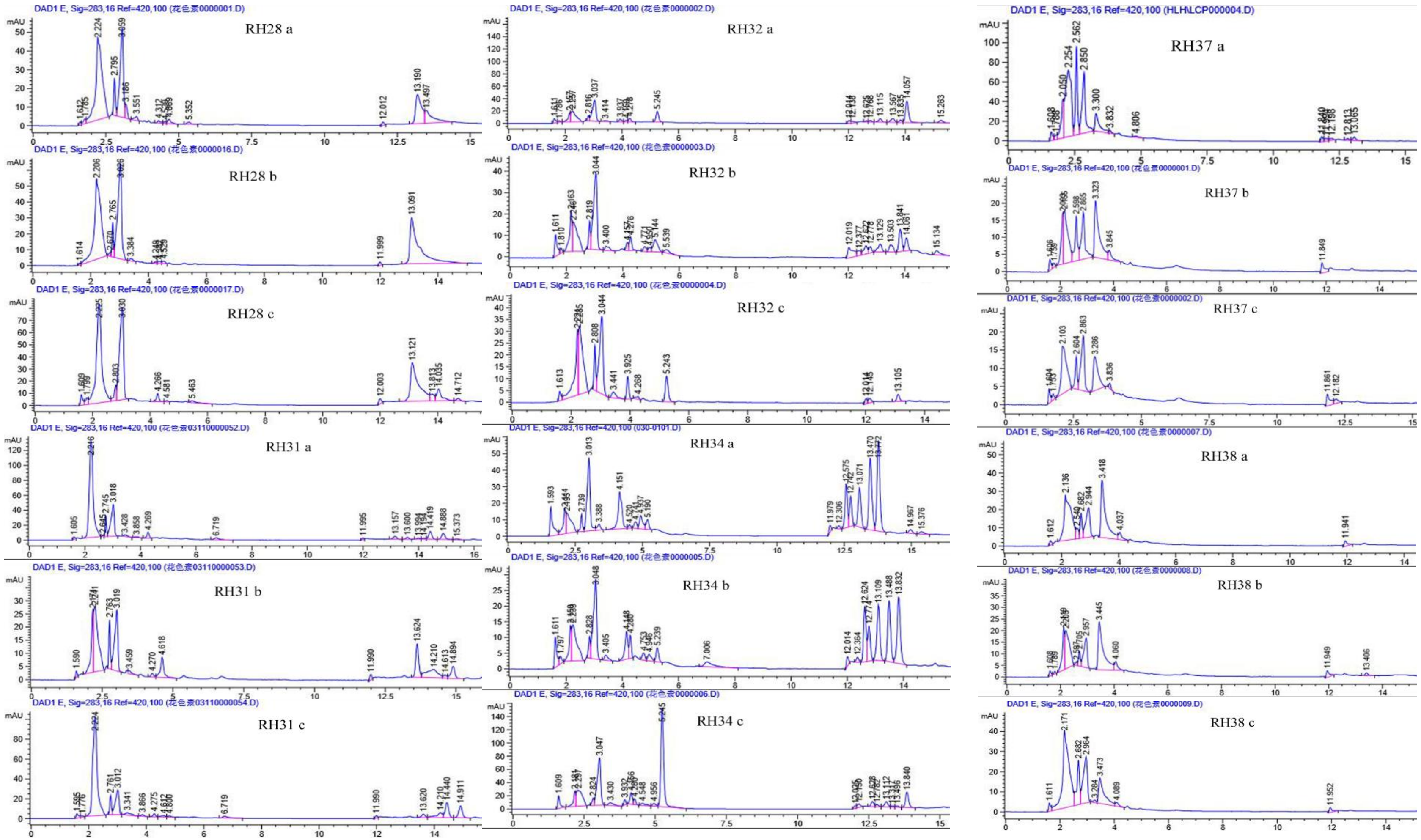
RH28 a
RH28 b
RH28 c
RH31 a
RH31 b
RH31 c
RH32 a
RH32 b
RH32 c
RH34 a
RH34 b
RH34 c
RH37 a
RH37 b
RH37 c
RH38 a
RH38 b
RH38 c

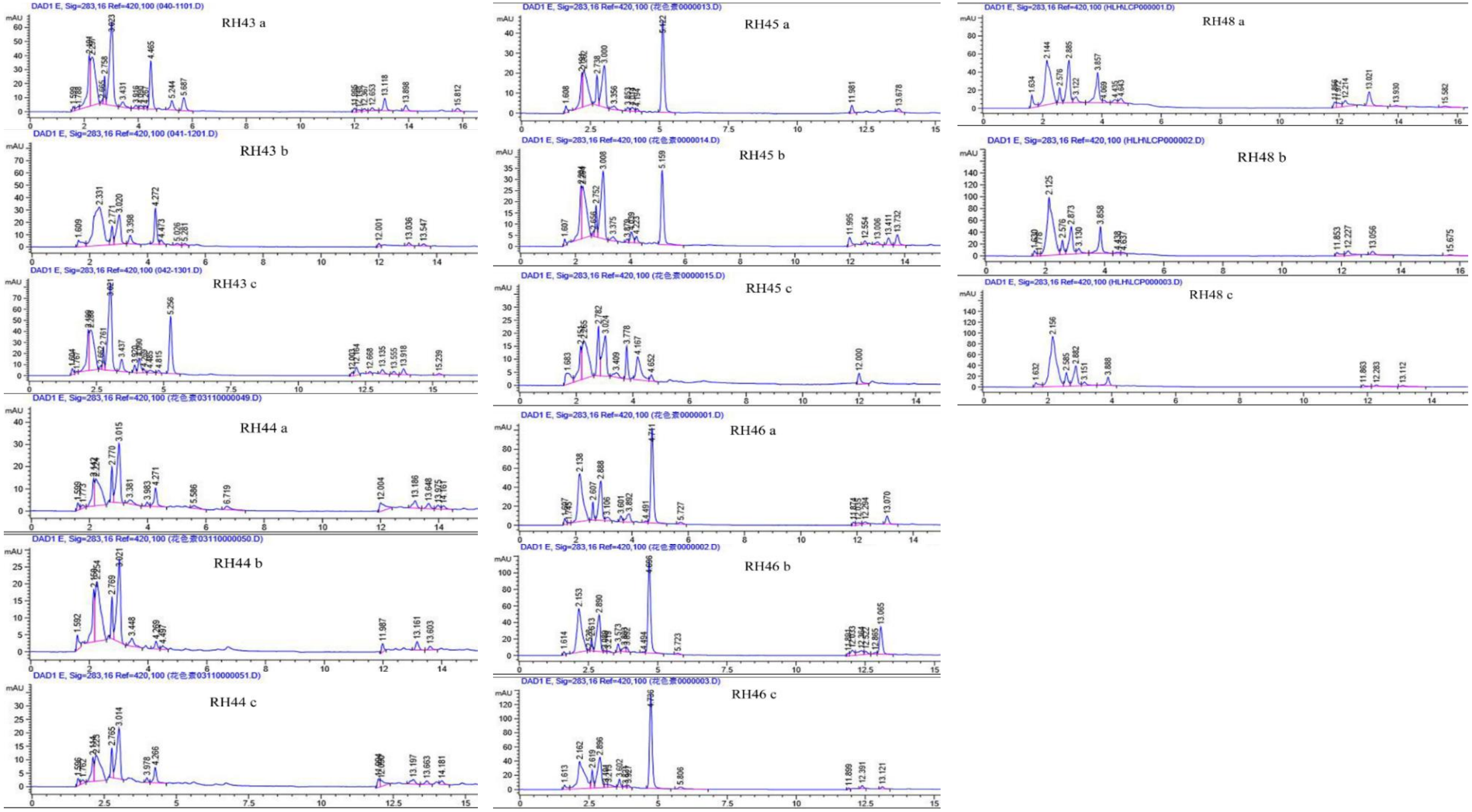

S1 Fig. HPLC chromatograms of all samples of grape cell extracts after co-cultured with different endophytic fungal strains (EFS).
