# Supplementary material for: Endophytic fungi specifically introduce novel metabolites into grape flesh cells in vitro
Source: PLoS One. 2018 May 7;13(5):e0196996. doi: 10.1371/journal.pone.0196996 (PMC5937782; doi:10.1371/journal.pone.0196996)
Supplement: S1 Table — (PDF) [file pone.0196996.s002.pdf]

S1 Table. Approaches gradient elution for HPLC

Time duration (min)	Elution solution (%)	Water (%)
0-2	35	65
2-5	15	85
5-8	20	80
8-15	40	60
15-20	35	65
