# Supplementary material for: Endophytic fungi specifically introduce novel metabolites into grape flesh cells in vitro
Source: PLoS One. 2018 May 7;13(5):e0196996. doi: 10.1371/journal.pone.0196996 (PMC5937782; doi:10.1371/journal.pone.0196996)
Supplement: S2 Table — (PDF) [file pone.0196996.s003.pdf]

S2 Table. HPLC detected metabolites and peak areas (mAU*S).

RT	2.25	2.72	2.99	3.37	3.59	3.78	3.99	4.28	4.5	4.8	5	5.14	11.9	12.1	12.3	12.5	12.8	13	13.5	13.8	14	14.4	14.97	15.2	15.6
Control	314	131	166	36								92	30												
RH6	967	75	260	17				37				24	17				23	48							
RH12	450	79	319			46	23			666			11				21	45							
RH28	868	78	576					22		12			15					560							
RH32	376	55	309	20			31	34				83	35	20			17	35	46	52	160			32	
RH34	322	44	400	32			109	41		41		373	19		21	112	70	128	168	258					
RH37	593	207	273	177			17						28	13											
RH38	460	87	178	276			28						21												
RH45	337	81	216	20		27	52	13				271	27							24					
RH46	647	86	390	28		45	44	10		762			15		40			107							
RH48	1176	128	363	65			235	19					42		61			67							16
CS2	553	60	488	47			410	104		76	135	837	49	116	28	84		41		359					68
CS11	342	55	137			46		6					16												
CS13	1100	220	361	46	872			114		22			31												
CS16	1204	71	621	15		9		16					26					1118			63	96	12		
RH5	384	59	405	44			87	35			35	216	26	41	10	29	10	29	18	164			24		
RH7	400	97	308	49			91	9				156	16	32						46					
RH16	594	73	611	81			144	57			81	184	105	17	23	156	118	128	322		264			36	
RH24	695	79	199	17				32					20						11			49	33		
RH31	926	81	259					24	40				11						47		60	73	74		
RH43	697	80	509	59			52	64	90			140	16	35				37	16	36					
RH44	248	52	198				11	38					27					23	13						
